# Design Principles Guided by DFT Calculations and High-Throughput Frameworks for the Discovery of New Diamond-like Chalcogenide Thermoelectric Materials

**DOI:** 10.1021/acsami.4c04120

**Published:** 2024-05-21

**Authors:** Adolfo E. Rosado-Miranda, Victor Posligua, Javier Fdez. Sanz, Antonio M. Márquez, Pinku Nath, Jose J. Plata

**Affiliations:** † Departamento de Química Física, Facultad de Química, Universidad de Sevilla, Seville 41012, Spain; ‡ Institute for Chemical Reaction Design and Discovery (WPI-ICReDD), Hokkaido University, Sapporo 060-0808, Japan

**Keywords:** thermoelectrics, chalcogenides, design principles, *ZT*, transport properties

## Abstract

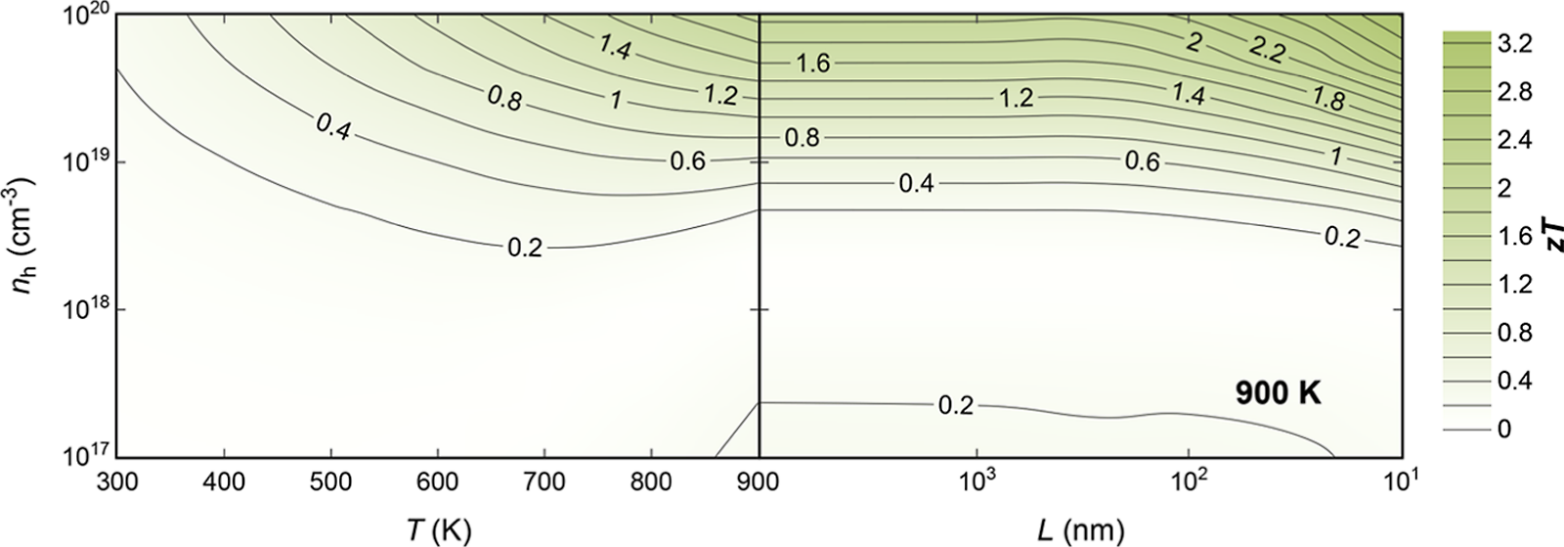

Rational design principles are one pathway to discovering new materials. However, technological breakthroughs rarely occur in this way because these design principles are usually based on incremental advances that seldom lead to disruptive applications. The emergence of machine-learning (ML) and high-throughput (HT) techniques has changed the paradigm, opening up new possibilities for efficiently screening large chemical spaces and creating on-the-fly design principles for the discovery of novel materials with desired properties. In this work, the approach is used to discover novel thermoelectric (TE) materials based on quaternary diamond-like chalcogenides. A HT framework that integrates density functional theory calculations, ML, and the solution of the Boltzmann transport equation is used to efficiently rationalize the transport properties of these compounds and identify those with potential as TE materials, achieving *ZT* values above 2.

## 1 Introduction

A large percentage of technological breakthroughs is sustained on materials discovery. Traditionally, there have been three main pathways to the synthesis of new materials.^1^ Some compounds are fortuitously discovered through serendipity, while pursuing different objectives. Others have been known for many years until their potential applications have been recognized and explored. The third pathway involves the use of design principles to predict and synthesize materials with well-defined properties. However, the emergence of high-throughput (HT), first-principles simulations and artificial intelligence (AI) has revolutionized the process of materials discovery and design.^2^ These methods facilitate the efficient screening of large compositional spaces, leading to the identification of novel compounds with targeted properties. Although these methods are often viewed as substitutes for chemical and physical intuition, they actually accelerate and enrich the development of new design principles.

While the extraction of materials design principles from the combination of HT calculations and machine learning (ML) has led to significant advances in many technological areas, this strategy faces significant challenges in the development of thermoelectric (TE) materials. Establishing design rules for materials applications with a single objective is trivial; however, the scenario is completely different when multiple and competing objectives are targeted.^3^ TE materials are particularly challenging due to the complex interplay of electrical conductivity (σ), thermal conductivity (κ), and Seebeck coefficient (*S*). Achieving high TE performance requires a delicate balance between these properties, which is often difficult to achieve through conventional materials design strategies. Additionally, the HT calculation of TE material properties as the main source for the application of AI/ML techniques presents severe limitations. The accurate prediction of electronic and thermal transport properties is several orders of magnitude more expensive than the other material properties linked to other technological applications.^4^ Additionally, transport properties strongly depend on many variables that can be modified during the synthesis and processing of the material but are not included in the simulation of bulk, stoichiometric, and defect-free materials.

During the past decade, the development of new methods and frameworks for the accurate prediction of thermal and electronic transport properties has accelerated the search for new and more efficient TE materials.^5,6^ Approaches based on the ML-aided extraction of high-order force constants have reduced the computational cost of simulating thermal transport properties without compromising accuracy.^7,8^ Similarly, a new whole family of packages has been developed for the accurate prediction of electronic transport properties,^9,10^ without the need for extensive computational resources required by the combination of Wannier functions and density functional pertubation theory (DFPT).^11^ For instance, Pal et al. have conducted an extensive screening of thousands of quaternary chalcogenides (AMM’X_3_) predicting their stability and computing the thermal conductivity of a reduced set of them.^12,13^

Chalcogenides are one of the largest family of compounds in the TE field due to their thermal and electronic features.^14^ Despite the large variety of compositions and structural prototypes, diamond-like (DL) compounds play a preponderant role due to their usually low cost and simple synthesis. DL chalcogenides are a rich set of materials in which all cations present tetrahedral coordination. Although binary DL chalcogenides have limited applications as TE materials, several high-performance alternatives can be found among ternary DL chalcogenides, such as CuFeS_2_,^15^ AgInSe_2_,^16^ and AgInTe_2_.^17^ However, quaternary DL chalcogenides are the best example of how these compounds can reach a figure of merit, *ZT*, of above 1.5. There are some examples in which the TE properties of some subset of quaternary DL chalcogenides have been explored guided by density functional theory (DFT) calculations.^18,19^ However, there is a need for design principles to effectively explore such an extensive chemical space.

In this work, we integrate HT frameworks and ML to accurately and systematically predict the lattice thermal conductivity of quaternary DL chalcogenides. These findings serve as a foundation for establishing a set of design rules to guide the search for new and more efficient TE materials. Through the application of these principles, we have not only explored but successfully optimized the TE performance of novel quaternary DL chalcogenides, surpassing *ZT* values of 2.

## 2 Results and Discussion

### 2.1 Validation

**Figure 1:**
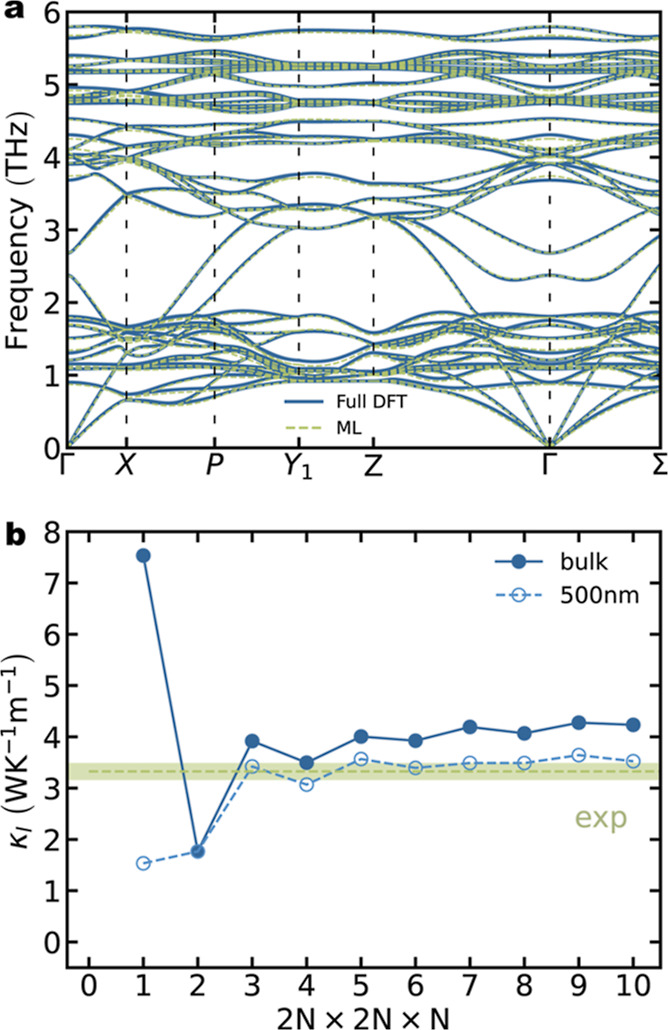
(a) Phonon dispersion curves for stannite Cu_2_CdSnTe_4_ using finite difference approach, full DFT (solid blue line) and ML regression, ML (dashed green line). (b) Lattice thermal conductivity, κ_l_, convergence with the density of the **q**-point mesh at 300 K. κ_l_ values are depicted for the Cu_2_CdSnTe_4_ single-crystal (blue solid line) and polycrystalline sample (dashed blue line). Green dashed line and green area represent reported experimental values.

While most of the methodology applied here has been validated in previous studies using DL ternary chalcogenides^20^ and pnictides,^21^ it is essential to verify the accuracy and reliability of our computational framework. Accurate determination of interatomic distances is crucial for obtaining precise κ_l_ values. The calculated lattice parameters are compared with experimental data for a selected group of materials to assess the efficacy of the DFT methodology in describing their structural properties. The calculated lattice parameters show a strong agreement with experimental values, with errors below 3% in all cases (Figure S1). For instance, the local density approximation (LDA) and the Perdew–Burke–Ernzerhof (PBE) functionals tend to underestimate and overestimate the experimental lattice parameter of Cu_2_CdSnTe_4_, respectively. In contrast, the PBE-D3 functional provides a much closer prediction to the experiment. Optimizing the calculation of κ_l_ from first principles involves considering factors beyond just the exchange–correlation functional. Interaction cutoff radii are used to reduce the computational costs. While harmonic (second-order) force constant cutoffs were selected based on the largest sphere that can be fit inside the supercell, third and fourth-order interatomic force constants (IFCs) were chosen according to previous study on ternary chalcogenides.^20^ A good agreement was achieved between phonon dispersion curves obtained using the finite-differences approach and the ML-aided methodology for Cu_2_CdSnTe_4_ (Figure 1a). The number of distorted supercells used in training the ML model can also modify the accuracy of the IFCs prediction, especially for third- and fourth-order force constants. It was found that using 30 supercells was sufficient to achieve a root mean squared error (RMSE) in forces below 22 meV/Å for the validation step as well as converge κ_l_ values (Figure S2). The calculated values of κ_l_ show a slight overestimation when compared to the experimental values reported for polycrystalline samples by Nolas et al.^22,23^ (Figure 1b). However, note that these calculated values do not take into account scattering processes caused by grain boundaries. Experimental reports have described the grains as large, with sizes exceeding 100 nm.^23^ If grain boundary scattering is included in the model, taking an average grain size of 500 nm, our predictions align much better with experimental results, showing less than a 5% error (Figure 1b).

### 2.2 Monovalent Atom Role

**Figure 2:**
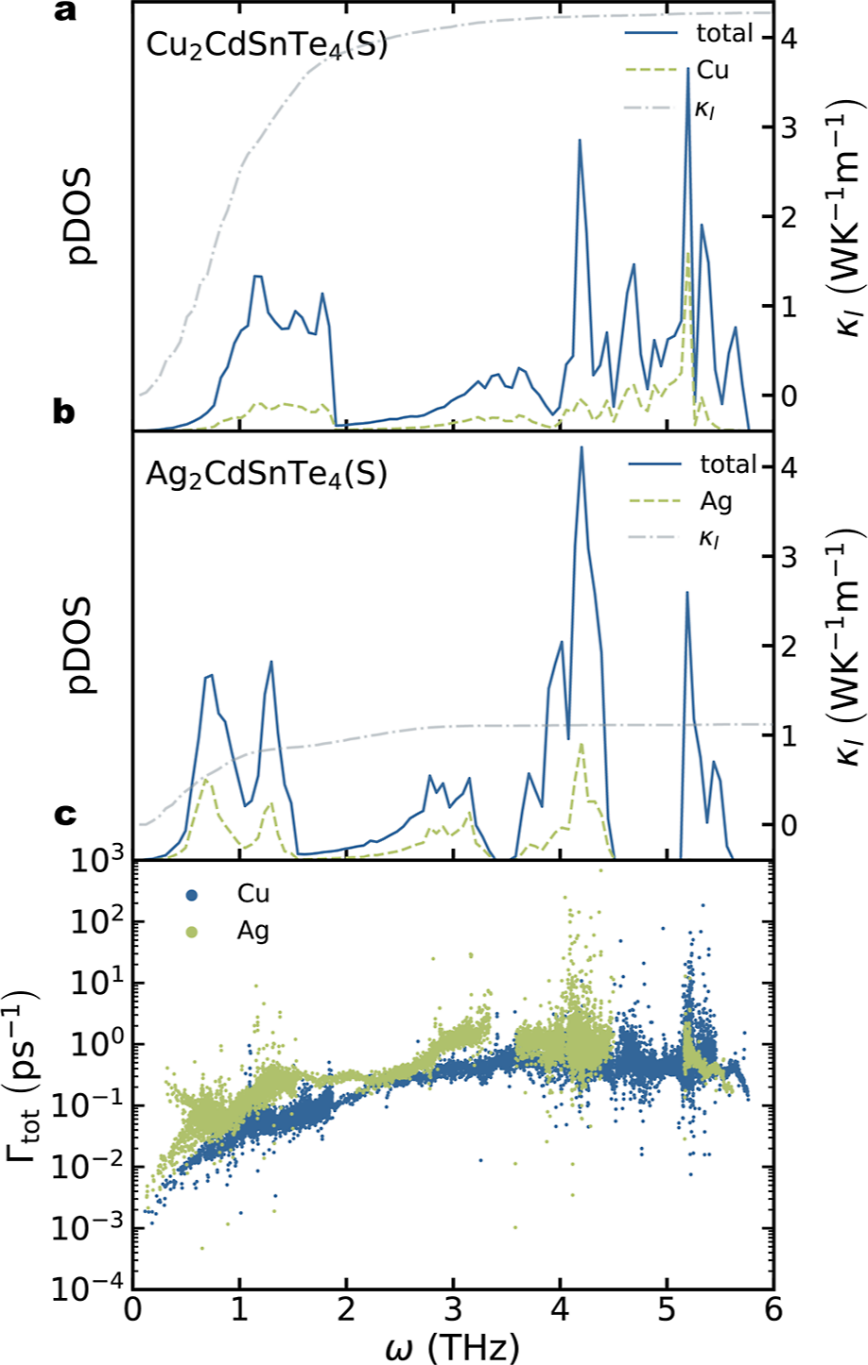
Phonon density of states (pDOS) for (a) Cu_2_CdSnTe_4_ and (b) Ag_2_CdSnTe_4_. Total pDOS is depicted using solid blue lines and the monovalent atom projection using dashed green lines. Cumulative lattice thermal conductivity as a function of phonon frequencies is included in dash-dotted grey lines. (c) Scattering rates vs mode frequency for Cu_2_CdSnTe_4_ (blue) and Ag_2_CdSnTe_4_ (green).

Extensive research has been focused on Cu-based chalcogenides due to their promising TE properties.^20,24^ However, it has been reported that Ag-based ternary chalcogenides present a significantly lower thermal conductivity than their Cu counterparts.^25^ Nevertheless, it remains unclear whether this trend can be generalized to other DL chalcogenides, and the mechanisms responsible for the reduced thermal conductivity in Ag-based chalcogenides are still not fully understood. To understand the role of the monovalent cation, the lattice thermal conductivity of Cu_2_CdSnTe_4_ and Ag_2_CdSnTe_4_ in their stannite structure was calculated. Phonon density of states (pDOS) already shows a clear difference between both solids due to the substitution of Cu by Ag (Figure 2a,b). The larger atomic mass of Ag compared to that of Cu significantly influences the contribution of the monovalent atom to the acoustic modes and low-energy optic modes. Ag-based compound presents an important contribution of the monovalent atom to the acoustic modes, while its contribution in the Cu counterpart is only noticeable for the low-energy optical modes. The substitution of Cu by Ag also introduces severe modifications in the anharmonicity of the materials through the phonon scattering rates, especially at low energies (Figure 2c). Ag_2_CdSnTe_4_ presents phonon scattering rates around 1 order of magnitude larger than those of Cu_2_CdSnTe_4_ in the low-energy range, anticipating lower lattice thermal conductivity values. Cumulative lattice thermal conductivity corroborates that the four times lower κ_l_ of the Ag-based compound is due to the higher scattering rates of both acoustic and low-energy optic modes (up to 1.75 THz). The origin of this difference in scattering rate values can be explained by comparing the phase space and the third-order IFCs of both compounds (Figure S3). Interestingly, Cu_2_CdSnTe_4_ presents larger values for the phase space than Ag_2_CdSnTe_4_, which would lead to higher scattering rates. This could be due to a more effective overlapping between vibrational modes in Cu_2_CdSnTe_4_ as observed in pDOS (Figure 2a,b). However, third-order IFCs are generally larger for Ag_2_CdSnTe_4_, explaining the larger scattering rates compared to the Cu-based compound.

### 2.3 Polymorphs

**Figure 3:**
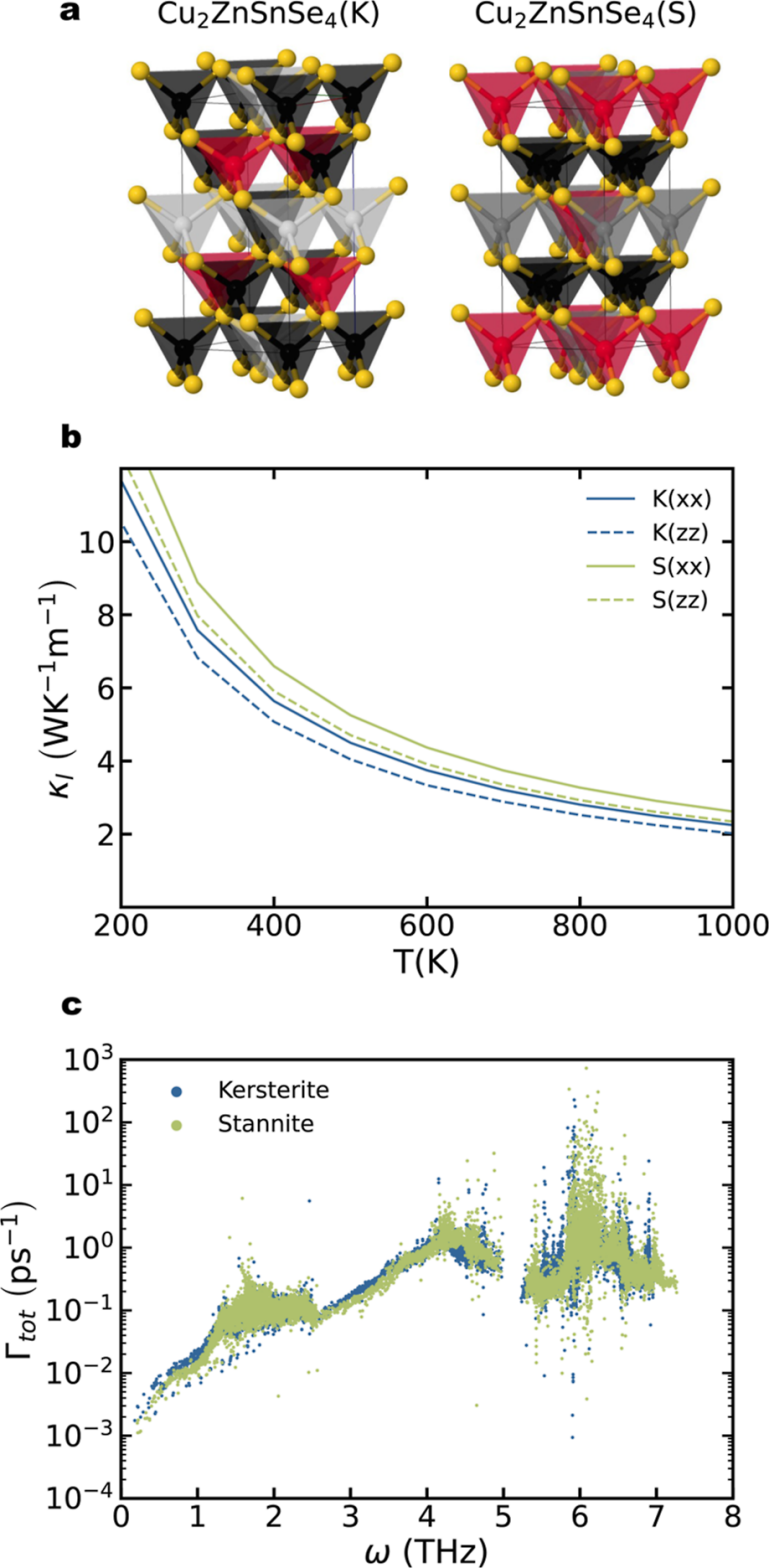
(a) Crystal structures for Cu_2_ZnSnSe_4_ kesterite (left) and stannite (right). Color code: black, Cu; gray, Sn; red, Zn; yellow, Se. (b) Lattice thermal conductivity as a function of temperature for Cu_2_ZnSnSe_4_ kesterite (blue) and stannite (green). Solid and dashed lines are used for in-plane (*xx*) and perpendicular (*zz*) κ_l_, respectively. (c) Scattering rates vs mode frequency for Cu_2_ZnSnSe_4_ kesterite (blue) and stannite (green).

One notable contrast between ternary and quaternary DL chalcogenides is the potential of several patterns or ordering motifs within the cation sublattice, which leads to the presence of different structural prototypes. In this work, all previous materials have been explored using the stannite polymorph (*I*4̅2*m*); however, it is worth noting that kesterite (*I*4̅) and primitive mixed CuAu-PMCA (*P*4̅2*m*) polymorphs have been also experimentally reported. Indeed, stannite and kesterite 0 K energies rarely differ by more than 10 meV/atom, making it challenging to differentiate them experimentally without advanced structural characterization techniques. To explore the role of different structural prototypes in the thermal properties of quaternary DL chalcogenides, the κ_l_ of Cu_2_ZnSnSe_4_ in both stannite and kesterite structures (Figure 3a) was calculated. The comparison of the pDOS for both polymorphs exhibited negligible differences (Figure S4). Both materials exhibit similar acoustic bands below 3 THz and two well-defined optic bands. The Cu_2_ZnSnSe_4_ stannite polymorph shows slightly higher lattice thermal conductivity compared to the kesterite structure in the whole temperature range (Figure 3b). This slight variation is attributed to the higher scattering rates of the acoustic band in the kesterite structure (Figure 3c). Approximately, 80% of the total thermal conductivity, κ_l_, in both compounds comes from vibrational modes with frequencies below 1.5 THz. The difference in thermal conductivity between the kesterite and stannite compounds can be attributed to their crystal symmetries. Kesterite space group is lower than stannite. This symmetry break promotes the appearance of vibrational modes with different frequencies, enhancing the number of scattering processes and reducing the κ_l_ value. Besides the difference between both polymorphs, it is worth noting the anisotropy of both compounds in terms of thermal conductivity. Both structural prototypes are constructed by stacking tetrahedra layers in the *z*-direction, creating a more homogeneous environment in the *xy*-plane. Consequently, κ_l_(*xx*) is consistently higher than κ_l_(*zz*) for both compounds.

### 2.4 Combining Design Principles

Based on the results presented in this study, optimizing κ_l_ to enhance *ZT* in quaternary DL chalcogenides is associated with two factors: (1) including Ag atoms and (2) synthesizing these compounds primarily in their kesterite form. However, a key challenge in maximizing *ZT* lies in the interconnection between the thermal and electronic transport properties of the material. For instance, the complete substitution of Cu by Ag in chalcogenides drastically reduces the carrier concentration in the compound, resulting in poor power factors (PF). Consequently, the presence of both Cu and Ag simultaneously has been reported in quaternary DL compounds, exhibiting outstanding performance.^26^

Stability and cost are two additional factors that should be considered beyond efficiency. Te-based compounds have been characterized in detail because of their high performance;^27,28^ however, the volatility of Te reduces their stability. Besides offering higher stability, the use of selenides instead of tellurides opens the door to a reduction in production costs. Selenium is approximately 50 times more abundant than Te in the Earth’s crust. Combining these design principles based on optimizing performance, stability, and production cost, the list of potential quaternary DL chalcogenides is drastically reduced. In this work, kesterite In_2_AgCuSe_4_ is proposed as a candidate as it fulfills all the aforementioned requirements.

**Figure 4:**
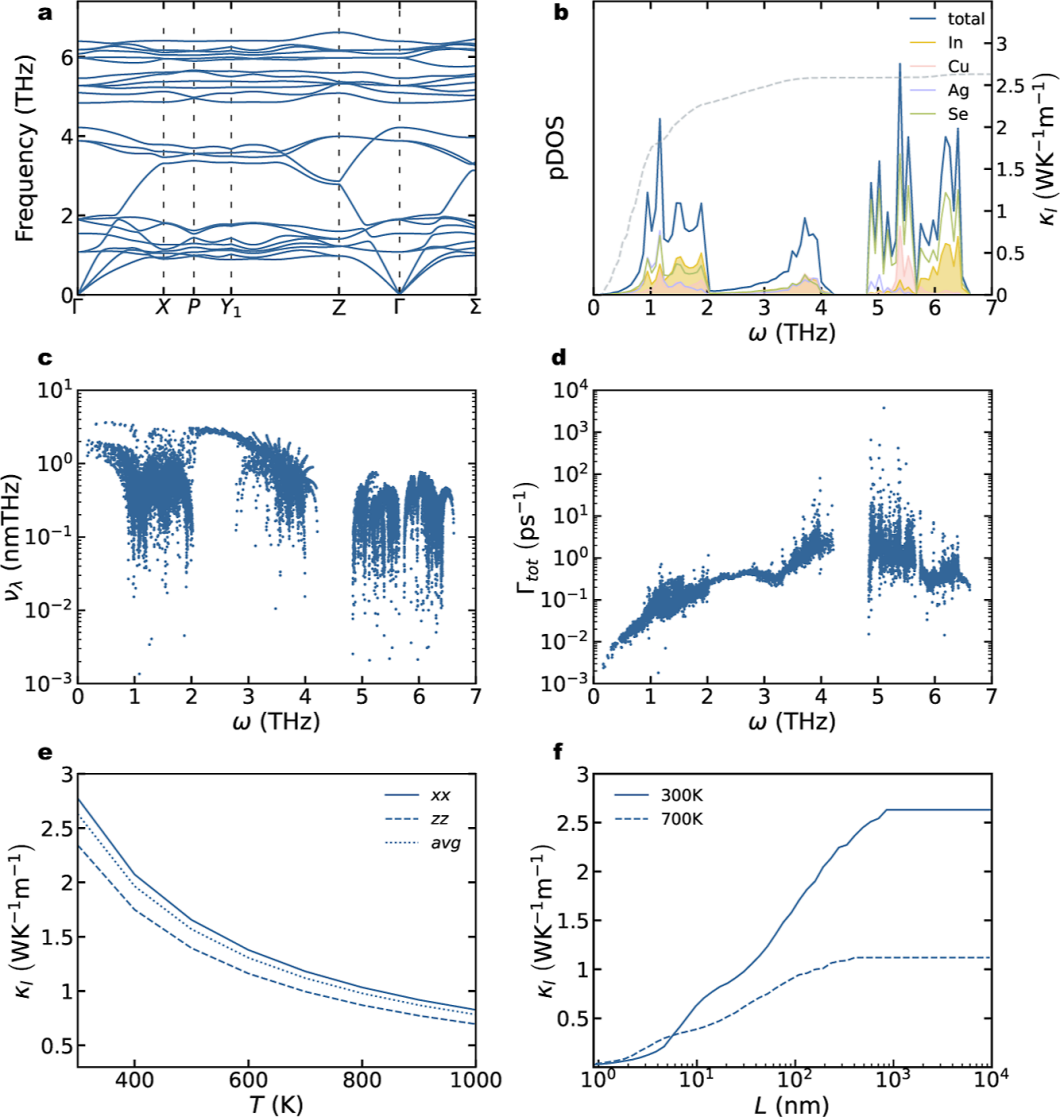
(a) Phonon dispersion curves for kesterite In_2_AgCuSe_4_. (b) Phonon density of states (pDOS) for kesterite In_2_AgCuSe_4_. Total pDOS is depicted using solid blue lines. In, Cu, Ag, and Se atom projections are depicted using yellow, red, purple, and green solid lines, respectively. Cumulative lattice thermal conductivity as a function of phonon frequencies is included in dashed grey lines. (c,d) In_2_AgCuSe_4_ group velocities and scattering rates as a function of phonon frequencies. (e) Lattice thermal conductivity as a function of temperature for In_2_AgCuSe_4_ kesterite. Solid, dashed, and dotted lines are used for in-plane (*xx*), perpendicular (*zz*), and average κ_l_, respectively. (f) Cumulative lattice thermal conductivity from mean-free-path contributions up to distance *L* for In_2_AgCuSe_4_, illustrating the effect that nanostructuring would have on thermal conductivity. Solid and dashed lines denote *T* = 300 and 700 K, respectively.

In_2_AgCuSe_4_ dispersion curves exhibit characteristics consistent with low thermal conductivity (Figure 4a). These features include the presence of low-lying optical modes and flat bands. Optical modes overlap with acoustic modes in the low-frequency region (around 1 THz), resulting in increased phonon scattering and decreased thermal conductivity. At 300 K, In_2_AgCuSe_4_ exhibits a lattice thermal conductivity of around 2.6 W/mK, with approximately 70% attributed to acoustic modes below 1 THz (Figure 4b). Projected pDOS shows that Ag, In, and Se are the main contributors to these low energy modes (Figure 4b). Flat dispersion bands indicate low group velocities. Only a few modes in the 0–1 and 2–3 THz intervals have group velocities greater than 1 nmTHz (Figure 4c). Large scattering rates, similar to the ones obtained for Ag_2_CdSnTe_4_, are a clear sign of the anharmonicity of the material (Figure 4d). The lattice thermal conductivity tensor as a function of the temperature is depicted in Figure 4e. The anisotropy of κ_l_, measured as κ_l_(*zz*)/κ_l_(*xx*), for this compound at 300 K is around 0.83. This value is significantly larger than the values reported for ternary chalcopyrites^20^ and closer to those of ternary pnictides.^21^ The lattice thermal conductivity is mainly influenced by phonon–phonon Umklapp scattering, resulting in a *T*^–1^ dependence (−1.075 when data is fitted). This reflects the growing number of phonons available for scattering as temperature increases. Lattice thermal conductivity can also be drastically reduced by nanostructuring. To quantify the effect of nanostructuring on κ_l_ reduction at a specific temperature, we calculated the particle size (or more precisely, the mean-free-path threshold) that results in a 50% decrease in the bulk value of lattice thermal conductivity, *L*_0.5_ (Figure 4f). Although In_2_AgCuSe_4_ presents low *L*_0.5_ values (below 100 nm for both 300 and 700 K), it is possible to reduce the κ_l_ of this compound around 20–40% by nanostructuring it to a particle size between 100 and 300 nm at both 300 and 700 K.

**Figure 5:**
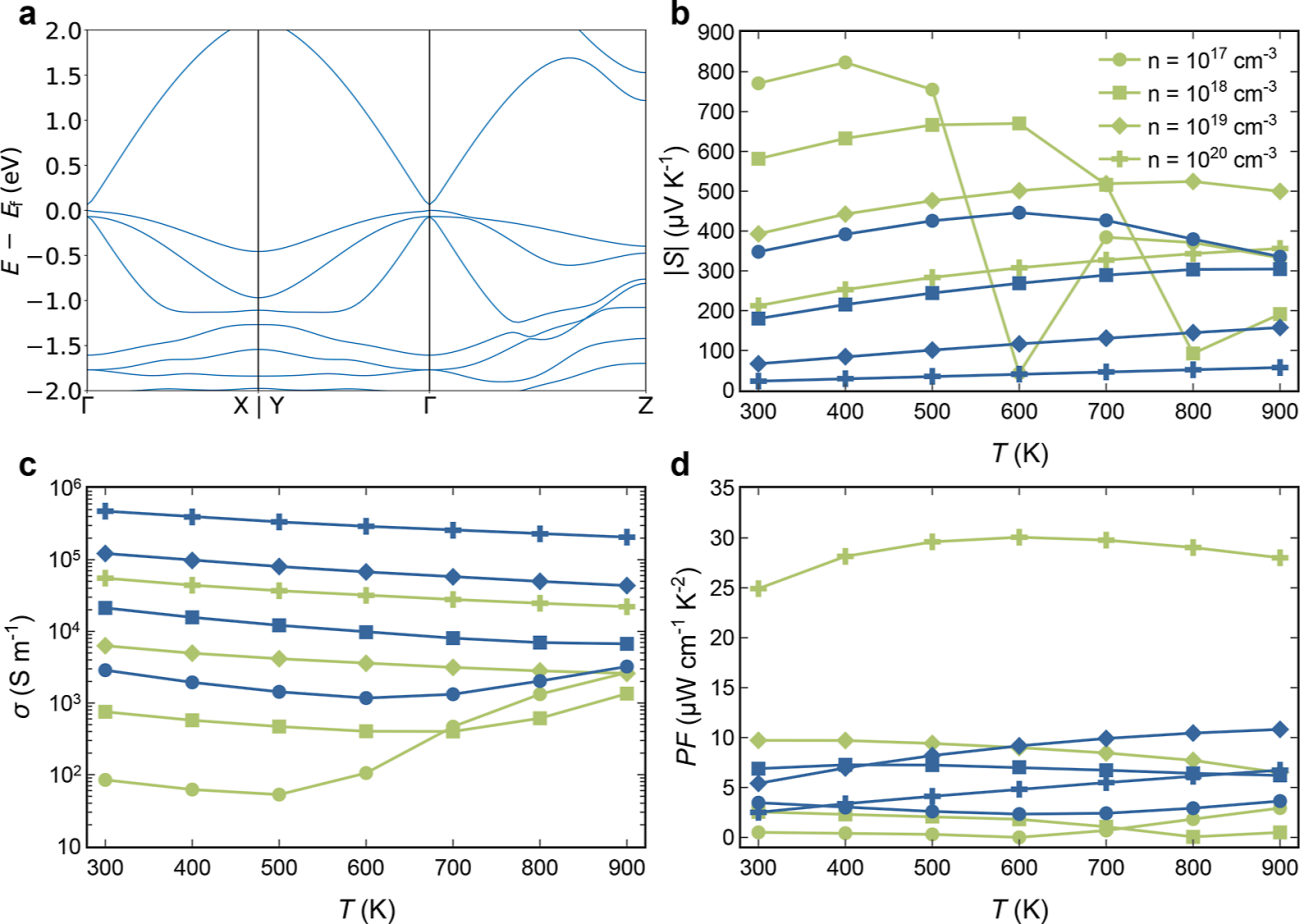
(a) Electronic band structure for In_2_AgCuSe_4_. (b–d) Seebeck coefficient (*S*), electrical conductivity (σ), and power factor (PF) for p- (green) and n-type (blue) In_2_AgCuSe_4_ as a function of temperature and carrier concentration, respectively.

Low lattice thermal conductivity alone is not sufficient to ensure high TE performance; electronic transport properties also play a critical role. The electronic band structure presents two main features that govern the electronic transport properties: the band gap and effective masses. The PBE functional predicts a band gap of less than 0.1 eV (Figure 5a), while the HSE functional obtained a gap of approximately 0.6 eV, in close agreement with the predicted band gaps for CuInSe_2_ and AgInSe_2_, which ranged between 0.5 and 1 eV.^29,30^ Due to significant differences in the topology of bands at the edge of the valence and conduction bands, variations should be observed between p- and n-type behavior of the material. The conduction band presents a Γ-centered main pocket with a relatively low effective mass, indicating high electron mobility, μ. However, the valence band presents different pockets close in energy at the Γ point. These pockets have different effective masses, indicating a more complex behavior for hole conductivity based on their occupancy. The qualitative predictions from the band structure are supported by the behavior of *S* and σ (Figure 5b,c). For n-type In_2_AgCuSe_4_, high mobility, moderate *S*, high σ, and moderate PF are observed (Figure 5d). However, p-type In_2_AgCuSe_4_ shows a more complex behavior, as anticipated by the band structure. At low hole concentrations and temperatures, this material exhibits a large Seebeck coefficient in agreement with the large effective masses and low mobility of states at the edge of the valence band (Figure 5b). However, bands with higher mobility near energy become populated at higher temperatures, leading to a drastic drop in *S* at higher temperatures and low carrier concentrations. This trend changes at higher carrier concentrations where more bands become populated at lower temperatures. The presence of quasi-degenerate states also enhances electrical conductivity, resulting in remarkable values of PF, especially at high carrier concentrations (Figure 5d).

Once the primary trends have been examined based on temperature and carrier concentration, it is essential to investigate the significance of each type of scattering mechanism incorporated in the theoretical model. This step is crucial for rationalizing how the electronic transport properties can be altered in In_2_AgCuSe_4_. An effective method for this purpose is to evaluate the contributions of each scattering mechanism to the carrier mobility (Figure S5). It looks like polar optical phonon (POP) scattering prevails as the dominant mechanism affecting carrier mobility in In_2_AgCuSe_4_ across all temperatures and carrier concentrations (Figure S5). The electrons are scattered through the interaction of the Coulomb field of the lattice polarization waves due to optical vibrational modes. Only at very high carrier concentrations, ionized impurity (IMP), and scattering processes contribute to total mobility for p-type In_2_AgCuSe_4_. This finding once again emphasizes a strong correlation between phonon vibrational structure and transport properties. While acoustic modes are crucial for thermal transport properties, optic modes also significantly influence electron mobility within this compound.

**Figure 6:**
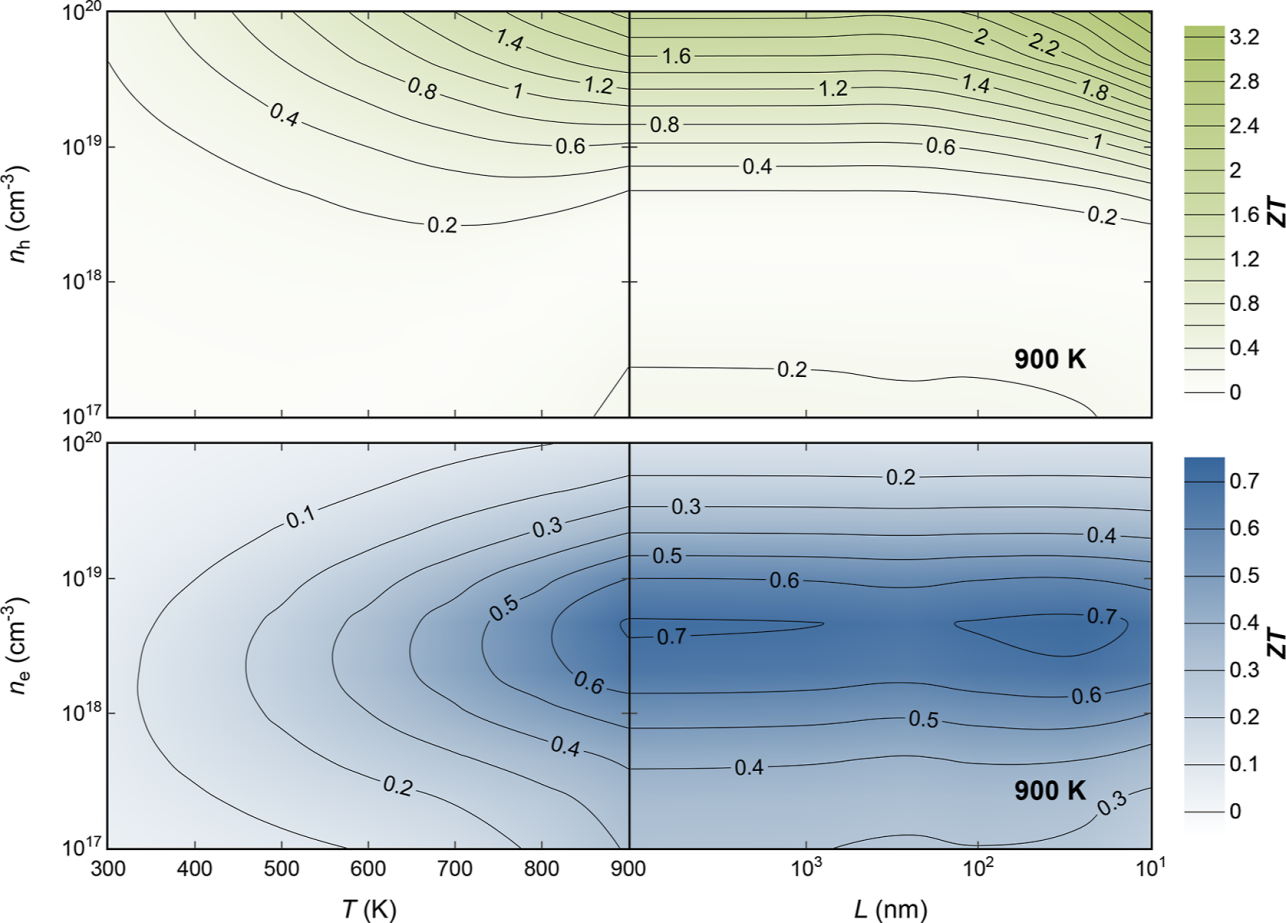
TE figure of merit, *ZT*, dependence on temperature, *T*, carrier concentration, *n*, and grain size, *L*, for p- (green) n-type (blue) In_2_AgCuSe_4_.

The TE figure of merit, *ZT*, for p-type and n-type In_2_AgCuSe_4_ has been calculated by combining κ and PF over a wide range of temperatures, *T*, and carrier concentrations, *n* (Figure 6). Similar to other DL chalcogenides, p-type In_2_AgCuSe_4_ exhibits higher *ZT* values compared to those of its n-type counterpart, particularly at high carrier concentrations. While the maximum *ZT* for bulk n-type In_2_AgCuSe_4_ is around 0.6–0.7 at moderate carrier concentrations and high temperatures, bulk p-type In_2_AgCuSe_4_ shows a maximum *ZT* of around 1.8 at high carrier concentration and temperature (Figure 6). This represents not only quantitative but also qualitative differences between both semiconductors especially in the TE optimization process. These differences stem from the distinct topology described at the edges of the conduction and valence band. The parabolic single band at the bottom of the conduction band makes temperature the main variable for maximizing *ZT* by reducing κ_l_. Nevertheless, the complex topology of the top of the valence band, featuring multiple pockets with similar energies, yet significantly different effective masses, opens up opportunities for *ZT* maximization. This can be achieved not only by reducing κ_*l*_ as the temperature increases but also by increasing hole concentration. To the best of our knowledge, there are no previous studies on the synthesis and carrier concentration of In_2_AgCuSe_4_. However, there are many reports about the dopability of CuInSe_2_ and AgInSe_2_. As expected, CuInSe_2_ presents higher carrier concentration levels (10^19^ cm^–3^ as p-type) than AgInSe_2_ (2.5 × 10^18^ cm^–3^ as n-type).^31^

Optimizing *ZT* requires taking into account factors beyond the temperature and carrier concentration. The microstructure plays a critical role in determining the TE properties of DL chalcogenides. Many strategies have been reported for the synthesis and processing of DL chalcogenides, resulting in substantial improvements in their TE performance. The methodology used in this study can consider the influence of grain boundaries on the electronic and thermal transport properties of materials. The primary aim is to optimize *ZT* through nanostructuring by identifying a grain size window where κ_l_ can be reduced without significantly reducing the PF. For instance, contour lines aligned parallel to the *x*-axis suggest that reducing the grain size, *L*, does not have a significant impact on *ZT*. This is the case for n-type In_2_AgCuSe_4_ (Figure 6). However, for p-type In_2_AgCuSe_4_, the contour lines show an improvement of the *ZT* values (above 2) with average grain sizes below 100 nm (Figure 6). This indicates that nanostructuring can effectively enhance the TE performance of p-type In_2_AgCuSe_4_ by reducing the thermal conductivity without compromising the PF.

## 3 Conclusions

The application of design principles in materials discovery has become increasingly powerful with the emergence of HT techniques and ML. These methods open doors to on-the-fly design principles while exploring large chemical and physical spaces. This is particularly interesting in fields where multiple and competing objectives are targeted, such as thermoelectricity. In this work, DFT calculations, ML techniques, and the use of the Boltzmann transport equation for phonons were combined into an HT framework to explore the TE performance of DL quaternary chalcogenides. This framework has been used to investigate the lattice thermal conductivity of these compounds and simultaneously establish design principles to accelerate the discovery of new TE materials. First, we discussed the critical role of substituting Cu for Ag to reduce the lattice thermal conductivity. Next, we explored the differences in thermal transport between various polymorphs and identified the structural prototype kesterite as the best candidate. By combining these design principles with other factors such as stability and cost-effectiveness, In_2_AgCuSe_4_ was proposed as a potential TE candidate. This material exhibits low lattice thermal conductivity (around 1 W/m K at 700 K) due to the scattering between acoustic and low-energy optical modes. The presence of different pockets with similar energies close to the top edge of the valence band makes In_2_AgCuSe_4_ an interesting p-type semiconductor with a large PF when the carrier concentration is around 10^20^ cm^–3^. While n-type In_2_AgCuSe_4_ presents a *ZT* maximum around 0.7, p-type In_2_AgCuSe_4_ can reach *ZT* values higher than 1.8 at 900 K. *ZT* can also be optimized by including the average grain size as a variable. For grain sizes around 100 nm, lattice thermal conductivity is drastically reduced, and PF is only slightly modified, resulting in *ZT* values larger than 2 for p-type In_2_AgCuSe_4_.

## 4 Methodology and Computational Details

### 4.1 Thermal Transport Properties

#### 4.1.1 Geometry Optimization

All ground state structures at 0 K were fully optimized (atoms and lattice parameters) using the VASP package,^32,33^ with projector-augmented wave potentials.^34^ The PBE exchange–correlation functional,^35^ along with Grimme D3 van der Waals correction,^36^ were combined to obtain the energies. Core and valence electrons were selected following standards proposed by Calderon et al.^37^ A high-energy cutoff of 500 eV and a dense **k**-point mesh of 2300 **k**-points per reciprocal atom were used. Convergence of the wave function was determined when the energy difference between consecutive electronic steps fell below 10^–9^ eV. The geometry and lattice vectors were relaxed until forces on all atoms reached values below 10^–7^ eV/Å, using a conventional cell containing 16 atoms. To minimize the noise in the forces, an additional support grid was included for the evaluation of the augmentation charges.

#### 4.1.2 Supercell Single-point Calculations and Force Constants

Interatomic force constants (IFCs) were calculated using the hiPhive package, which combines the forces calculated for distorted atoms in supercells with ML regression.^38^ A 3 × 3 × 2 supercell containing 288 atoms was used for the calculation of forces, following the same setup as the geometry optimizations. To ensure the accurate calculation of the IFCs, a two-step approach was implemented.^20^ First, small random distortions were applied to all atoms in three supercells, and then second- and third-order IFCs were extracted using the hiPhive package. Subsequently, an additional set of 18 distorted supercells was generated by superimposing normal modes with random phase factors and amplitudes corresponding to a temperature of 300 K by employing the second-order IFCs obtained from the previous step. To maximize the use of data and minimize training bias, we incorporated 5-fold cross-validation into our workflow. This involved training the model on 80% of the data while rotating through different subsets for validation at each fold. The IFCs were determined using the recursive feature elimination (RFE) algorithm through multilinear regression to the DFT forces. Despite being more computationally expensive compared to ordinary least-squares regression, RFE has shown its effectiveness in achieving convergence with fewer structures.^8^ Moreover, employing an RFE reduces the number of parameters and simplifies the model by retaining only the most significant interaction terms. IFCs were calculated including cutoffs for second-, third-, and fourth-order terms. To ensure transferability across different compounds, these cutoff values were determined based on coordination shells.

#### 4.1.3 BTE Solver

The lattice thermal conductivity, κ_l_, is calculated using the ShengBTE code, through the iterative solution of the BTE for phonons.^39^ This method provides more accurate results compared with the relaxation time approximation. Scattering rates are computed by considering isotopic and three-phonon scattering processes. To balance memory demand and ensure convergence of κ_l_ with respect to **q**-points, a Gaussian smearing of 0.1 is applied along with a dense mesh of 16 × 16 × 8 **q**-points.

### 4.2 Electronic Transport Properties

The AMSET package,^9^ consistent with the methodology outlined in our previous study,^17^ was employed to calculate the electrical conductivity, the Seebeck coefficient, and the electronic contribution to thermal conductivity. This tool solves the Boltzmann transport equation to predict electronic transport properties using Onsager coefficients, with the wave function derived from a DFT calculation serving as the primary input. Scattering rates for each temperature, doping concentrations, bands, and **k**-points were determined by considering scattering due to deformation potentials, POPs, and ionized impurities. Wave function coefficients were calculated using the HSE06 functional^40,41^ employing the primitive cell (8 atoms) and a 12 × 12 × 12 **k**-point mesh. Elastic constants and deformation potential, crucial to calculating different scattering contributions, were obtained following the same configuration used for geometry optimization and force constant calculations.

## Supporting Information Available

Lattice parameters comparison, validation RMSE in the forces, phonon density of states, and electron mobility. Further data sets are available at the ZENODO repository (doi: 10.5281/zenodo.10687721) (PDF)
