# Supplementary material for: Design Principles Guided by DFT Calculations and High-Throughput Frameworks for the Discovery of New Diamond-like Chalcogenide Thermoelectric Materials
Source: ACS Appl Mater Interfaces. 2024 May 21;16(22):28590–8. doi: 10.1021/acsami.4c04120 (PMC11163396; doi:10.1021/acsami.4c04120)
Supplement: Supplementary file 1 — am4c04120_si_001.pdf [file am4c04120_si_001.pdf]

Supporting Information
Design Principles Guided by DFT Calculations and High-Throughput Frameworks for the Discovery of New Diamond-Like Chalcogenide Thermoelectric Materials

Adolfo E. Rosado-Miranda,[†,¶] Víctor Posligua,[†,¶] Javier Fdez. Sanz,[†] Antonio M. Márquez,[†] Pinku Nath,[*,‡] and Jose J. Plata[*,†]

†*Departamento de Química Física, Facultad de Química, Universidad de Sevilla, Seville 41012, Spain*

‡*Institute for Chemical Reaction Design and Discovery (WPI-ICReDD), Hokkaido University, Sapporo, Japan*

¶*These authors contributed equally to this work*

E-mail: pnath@icredd.hokudai.ac.jp; jplata@us.es

Abstract

Rational design principles are one pathway to discovering new materials. However, technological breakthroughs rarely occur in this way because these design principles are usually based on incremental advances that seldom lead to disruptive applications. The emergence of machine learning and high-throughput techniques has changed the paradigm, opening up new possibilities for efficiently screening large chemical spaces

and creating on-the-fly design principles for the discovery of novel materials with desired properties. In this work, the approach is used to discover novel thermoelectric materials based on quaternary diamond-like chalcogenides. A high-throughput framework that integrates DFT calculations, machine learning, and the solution of the Boltzmann transport equation is used to efficiently rationalize the transport properties of these compounds and identify those with potential as thermoelectric materials, achieving ZT values above 2.

Keywords

Thermoelectrics, chalcogenides, design principles, ZT, transport properties

1 Validation

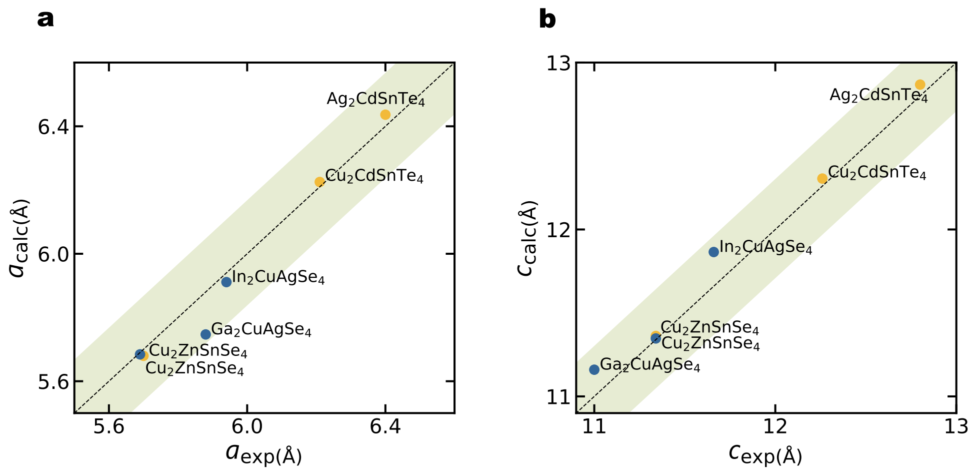

Figure S1: Comparison of DFT-calculated vs experimental cell parameters **a** a and **b** c. The dashed line in each plot represents perfect agreement, and the green shaded area represents deviations of 2% from experiment in either direction.

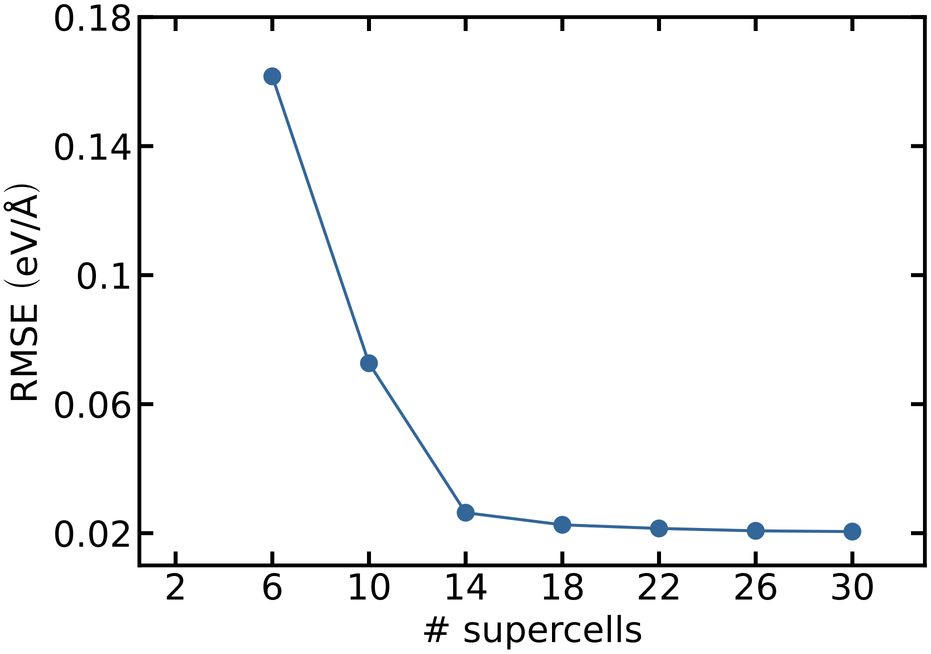

Figure S2: Validation RMSE in the forces for stannite $Cu_2CsSnTe_4$ as function of the number of supercells used in the training.

2 Ag and Cu compounds

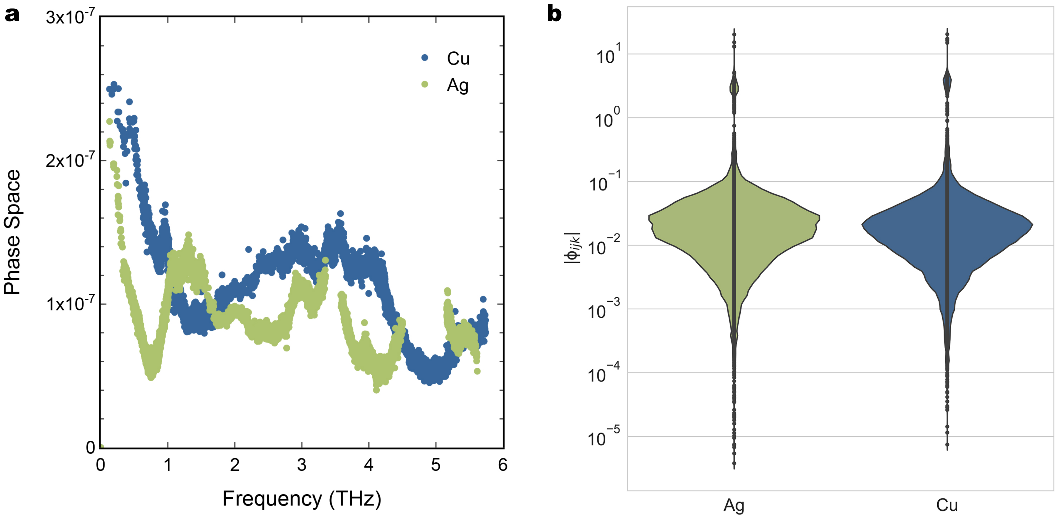

Figure S3: **a** Phase Space and **b** third-order IFCs, Φ_{ijk}, for $Cu_2SnCdTe_4$ (blue) and $Ag_2SnCdTe_4$ (green).

3 Polymorphs

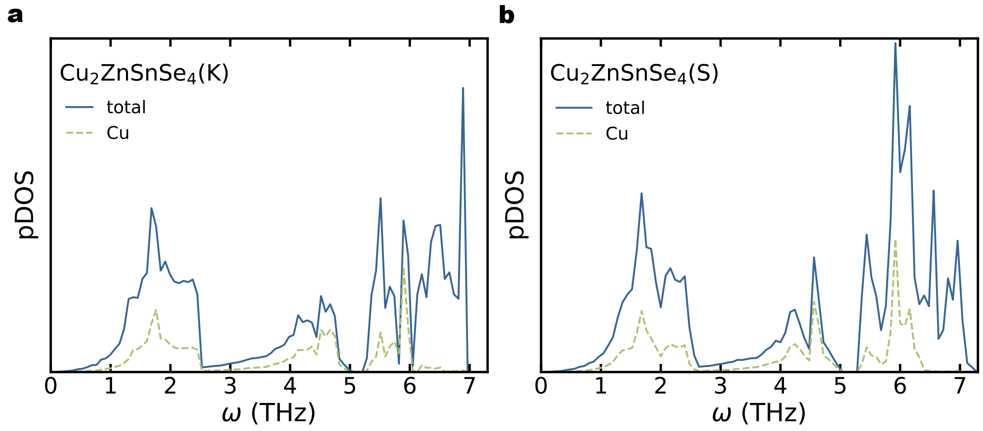

Figure S4: Phonon density of states, pDOS, for **a** kesterite and **b** stannite $Cu_2ZnSnSe_4$. Total pDOS is depicted using solid blue lines and the Cu atom projection using dashed green lines.

4 Electronic transport properties - AMSET

AMSET code solves the BTE for electrons without the RTA. Scattering rates are calculated using the Matthiesen's rule:

$$\frac{1}{\tau_e} = \frac{1}{\tau^{\mathrm{ADP}}} + \frac{1}{\tau^{\mathrm{IMP}}} + \frac{1}{\tau^{\mathrm{POP}}} + \frac{1}{\tau^{\mathrm{MFP}}}, \tag{1}$$

where τ^{ADP}, τ^{IMP}, τ^{ADP} and τ^{MFP} represent the scattering times due to the acoustic deformation potential, ionized impurities, polar optical phonons and grain boundaries, respectively. Piezoelectric scattering has not been included due to the centrosymmetric nature of these materials. The mode-dependent scattering rates, from state $|n\mathbf{k}\rangle$ to state $|m\mathbf{k}+\mathbf{q}\rangle$, are calculated using the Fermi's golden rule:

$$\tilde{\tau}^{-1}_{n\mathbf{k}\to m\mathbf{k}+\mathbf{q}} = \frac{2\pi}{\hbar} \left|g_{nm}(\mathbf{k},\mathbf{q})\right|^2 \delta\left(\varepsilon_{n\mathbf{k}} - \varepsilon_{m\mathbf{k}+\mathbf{q}}\right) \tag{2}$$

with ε being the electron energy, δ the Dirac delta function, and g the coupling matrix element. Electron transport properties were computed by the generalized transport coefficients,

$$L^n_{\alpha\beta} = e^2 \int \sum_{\alpha\beta}(\varepsilon)\left(\varepsilon - \varepsilon_F\right)^n \left[-\frac{\partial f^0}{\partial \varepsilon}\right] d\varepsilon, \tag{3}$$

where α and β represent cartesian coordinates, $\sum_{\alpha\beta}(\varepsilon)$ is the spectral conductivity, ε_F is the Fermi level at a doping concentration and temperature and f^0 is the Fermi-Dirac distribution. Finally, electronic transport properties are calculated as,

$$\sigma_{\alpha\beta} = L^0_{\alpha\beta}, \tag{4}$$

$$S_{\alpha\beta} = \frac{1}{eT}\frac{L^1_{\alpha\beta}}{L^0_{\alpha\beta}}, \tag{5}$$

$$\kappa_{\alpha\beta} = \frac{1}{e^2T}\left[\frac{\left(L^1_{\alpha\beta}\right)^2}{L^0_{\alpha\beta}} - L^2_{\alpha\beta}\right]. \tag{6}$$

Required material parameters for the calculation of scattering times are obtained through DFT calculations. Dense uniform band structure and wave function coefficients are obtained through single point calculations of the fully relaxed primitive cells. Wavefunction was considered converged when the energy difference between two consecutive electronic steps was smaller than 10^{-9} eV, using a dense mesh of $12 \times 12 \times 12$ **k**-points and the HSE06 functional proposed by Heyd *et al.*[1] Deformation potential, $\mathbf{D}_{n\mathbf{k}}$, is calculated as,

$$\mathbf{D}_{n\mathbf{k}} = \delta\varepsilon_{n\mathbf{k}}/\delta S_{\alpha\beta}, \tag{7}$$

where $\mathbf{S}$ is the uniform stress tensor and $\varepsilon_{n\mathbf{k}}$ is the energy of a band in a specific **k**-point. The deformation potential is averaged over contraction (–0.5 %) and expansion (+0.5 %) of the lattice and calculated separately for each component of the strain tensor. Eigenvalues are aligned to the average energy level of the core states which are calculated using the initial-state approximation.[2,3] Calculated dielectric constants and effective polar phonon frequency are obtained using density functional perturbation theory (DFPT)[4,5] using the PBE functional.[6] Effective phonon frequency is determined from the phonon frequencies and phonon eigenvectors. To capture scattering from the full phonon band structure in a single phonon frequency, each phonon mode is weighted by the dipole moment.

Transport properties calculations were conducted under p- and n-type doping conditions within a range from 10^{17} to 10^{20} cm^{-3}, and the temperature range from 300 to 1000 K. Ionized impurity scattering was calculated using a charge of ±1 for the impurity. In order to ensure converged properties, interpolation factor was set to 50 for all calculations and the energy cutoff used to determine which bands to include in the interpolation and scattering rate calculation was set to 1.5 eV.

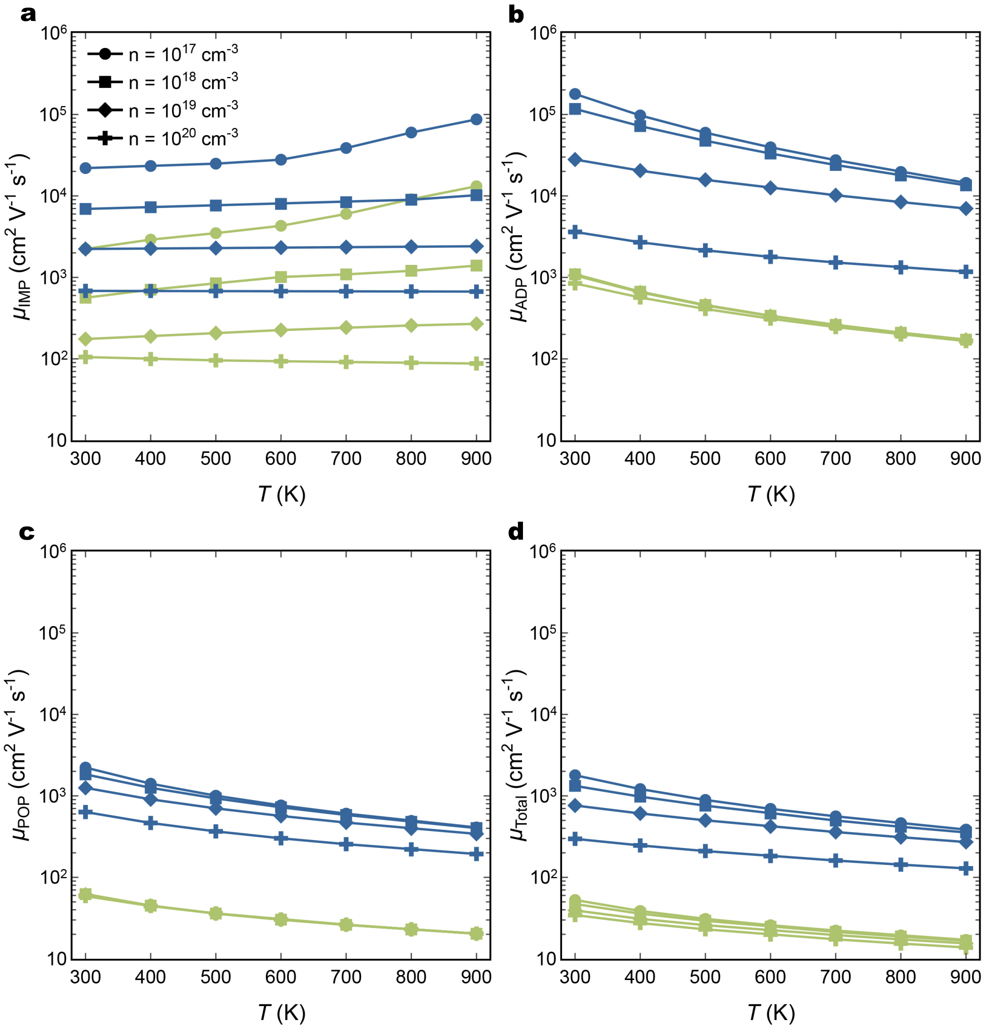

Figure S5: Electron (blue) and hole (green) mobility as function of temperature and carrier concentration. Panels **a**, **b**, **c** represent the contributions to mobility of IMP, ADP and POP scattering mechanism. Total carrier mobility is depicted in panel **d**

References

(1) Heyd, J.; Scuseria, G. E.; Ernzerhof, M. Erratum: "Hybrid Functionals Based on a Screened Coulomb Potential" [J. Chem. Phys.118, 8207 (2003)]. *J. Chem. Phys.* **2006**, *124*.

(2) Lizzit, S.; Baraldi, A.; Groso, A.; Reuter, K.; Ganduglia-Pirovano, M. V.; Stampfl, C.; Scheffler, M.; Stichler, M.; Keller, C.; Wurth, W.; Menzel, D. Surface Core-Level Shifts of Clean and Oxygen-Covered Ru(0001). *Phys. Rev. B* **2001**, *63*, 205419.

(3) Köhler, L.; Kresse, G. Density Functional Study of CO on Rh(111). *Phys. Rev. B* **2004**, *70*, 165405.

(4) Baroni, S.; Resta, R. Ab Initio Calculation of the Macroscopic Dielectric Constant in Silicon. *Phys. Rev. B* **1986**, *33*, 7017–7021.

(5) Gajdoš, M.; Hummer, K.; Kresse, G.; Furthmüller, J.; Bechstedt, F. Linear Optical Properties in the Projector-Augmented Wave Methodology. *Phys. Rev. B* **2006**, *73*, 045112.

(6) Hammer, B.; Hansen, L. B.; Nørskov, J. K. Improved Adsorption Energetics Within Density-Functional Theory Using Revised Perdew-Burke-Ernzerhof Functionals. *Phys. Rev. B* **1999**, *59*, 7413–7421.
